# Clustering of disability pension and socioeconomic disadvantage in Sweden: a geospatial analysis

**DOI:** 10.1093/eurpub/ckac096

**Published:** 2022-07-29

**Authors:** Marianna Virtanen, Katriina Heikkilä, Jussi Vahtera, Mika Kivimäki, Jaana I Halonen, Kristina Alexanderson, Simo Rautiainen, Tea Lallukka, Ellenor Mittendorfer-Rutz

**Affiliations:** Division of Insurance Medicine, Department of Clinical Neuroscience, Karolinska Institutet, Stockholm, Sweden; School of Educational Sciences and Psychology, University of Eastern Finland, Joensuu, Finland; Division of Insurance Medicine, Department of Clinical Neuroscience, Karolinska Institutet, Stockholm, Sweden; Finnish Institute for Health and Welfare, Helsinki, Finland; Department of Public Health, University of Turku and Turku University Hospital, Turku, Finland; Clinicum, Department of Public Health, University of Helsinki, Helsinki, Finland; Department of Epidemiology and Public Health, University College London, London, UK; Finnish Institute of Occupational Health, Helsinki, Finland; Finnish Institute for Health and Welfare, Helsinki, Finland; Department of Psychology, Stockholm University, Stockholm, Sweden; Division of Insurance Medicine, Department of Clinical Neuroscience, Karolinska Institutet, Stockholm, Sweden; Faculty of Social Sciences and Business Sciences, Karelian Institute, University of Eastern Finland, Joensuu, Finland; Division of Insurance Medicine, Department of Clinical Neuroscience, Karolinska Institutet, Stockholm, Sweden; Clinicum, Department of Public Health, University of Helsinki, Helsinki, Finland; Division of Insurance Medicine, Department of Clinical Neuroscience, Karolinska Institutet, Stockholm, Sweden

## Abstract

**Background:**

To characterize geospatial patterning of disadvantage in Sweden, we examined whether municipal-level indicators of socioeconomic disadvantage and disability pension (DP) rate were clustered, whether the different geospatial clusters were overlapping and whether the findings were similar among women and men.

**Methods:**

Administrative national data from all 290 Swedish municipalities were used to determine the prevalence of DP and socioeconomic disadvantage [poverty, long-term unemployment, income inequality (GINI Index) and income inequality between women and men]. Geospatial cold spots (clusters of municipalities with a DP/socioeconomic disadvantage prevalence lower than the nationwide prevalence) and hot spots (clusters of municipalities with a DP/socioeconomic disadvantage higher than the nationwide prevalence) were identified, and whether a hot spot was overlapping with another hot spot and a cold spot overlapping with another cold spot were analysed using the Getis-Ord Gi statistics.

**Results:**

Among women and men, cold spots of DP were most consistently located in the Stockholm area. Hot spots of DP were found in the mid-south Sweden, characterized by mid-sized urban centres in rural territories. High DP rate and socioeconomic disadvantage were overlapping, except for income inequality. Clusters of gender income inequality and women’s high DP rate were observed in mid-south Sweden.

**Conclusion:**

DP and socioeconomic disadvantage are not randomly distributed in Sweden. Geospatial analyses revealed clusters of municipalities with high risk of both DP and socioeconomic disadvantage in certain areas and low risk in other areas. Further research is needed to identify preventive actions to decrease regional inequalities in work capacity.

## Introduction

Early exit from the labour market due to work incapacity is a common challenge in high-income countries. In Sweden, for example, a total of 345 000 individuals received disability pension (DP) benefits in 2015,^1^ the prevalence (6%) being at the Organisation for Economic Co-operation and Development (OECD) average level.^2^ DP has traditionally been viewed as a consequence of an interplay between individual’s poor health status and his/her incapacity for work in the current or respective job. More recently, approaches taking into account impacts from various levels and contexts, such as working and living conditions and the macroeconomic level, have been developed.^3^ In Sweden, all people registered as living in Sweden and aged 19–64 could be granted DP if their work capacity is reduced permanently due to disease or injury. Among people aged 19–29 years, DP could also be granted if the work capacity reduction is long-term (e.g. for at least one year). The work incapacity could be granted for full-time (100%) or part-time (75%, 50% or 25% of ordinary work hours).

Research has shown that physically^4^^,^^5^ or mentally^6^ strenuous work and low socioeconomic status^7–9^ are associated with higher DP incidence. The impact of other contextual factors, such as geographic location, is poorly understood. High municipal-level unemployment rate has been shown to been associated with higher DP rates^10^^,^^11^ and long-term sickness absence.^12^ In Sweden, large old industrial areas, mainly in the rural and semi-urban part of the country have suffered from the adverse impacts of globalization, structural changes in the economy and downsizing of industries. Municipalities in Sweden are further grouped under 21 county councils which are responsible for primary and specialized healthcare. This may indicate clustering of DP beyond single municipalities.

Newly developed geospatial analysis techniques could help identify risky circumstances, such as place of residence. In Sweden, the DP rates are lower in the county of Stockholm than in the rest of Sweden,^7^ high-prevalence ‘hot spots’ have been found of intellectual disability in northern Sweden,^13^ high-incidence cardiovascular hot spots in northern and central Sweden^14^ and a lower prevalence of pre-pregnancy overweight/obesity in Stockholm area and in south and southwest Sweden than in other parts of the country.^15^ As there is a lack of geospatial analysis of clustering of DP in Sweden, it is not known whether DP is clustered in certain areas, whether those clusters overlap with other indicators of socioeconomic disadvantage and whether there are differences between women and men in this issue. In the present study, we assessed geospatial patterning of DP and socioeconomic disadvantage at the municipal level in Sweden. By increasing knowledge on geospatial patterns of clustered disadvantage we hope to provide information that helps targeting interventions and social and health services to high-risk areas.

## Methods

### Data sources

Information of DP among working aged (19–64 years) residents in Sweden was obtained from Micro-Data for Analysis of the Social Insurance System (MiDAS) kept by the National Social Insurance Agency,^16^ and the number of residents aged 19–64 years in each municipality was obtained from Longitudinal Integrated Database for Health Insurance and Labour Market Studies (LISA) kept by Statistics Sweden. Using these data and the municipality codes, aggregated age-adjusted prevalence of DP in 2015, separately for women and men, was calculated for each of Sweden’s 290 municipalities. The DP status was defined as having DP as one’s main activity during 2015.

An open-access database Kolada for municipal statistics of Sweden (www.kolada.se) was used to derive municipal-level socioeconomic indicators for 2015. Kolada is coordinated by the Council for the Promotion of Municipal Analyses (RKA), which is a non-profit association formed in collaboration between the state and the Swedish municipalities and regions (SKR). The open-access database contains data from national statistical authorities of Sweden aggregated at the municipal level. The following indicators of socioeconomic adversity in 2015 were derived: long-term unemployment rate among residents aged 25–64 years (those who in March 2015 had been unemployed or in employment programmes for at least 6 months), expressed as a proportion (%) of all residents on 31 December 2015 (Source: Swedish Public Employment Service); income inequality, which was based on the residents’ earned income and expressed as a Gini Coefficient which can vary between 0 (total equality/all households have same income) and 1 (total inequality/one household has all the income) (Source: Statistics Sweden); gender income inequality was an indicator of women’s median net income as a share of men’s median net income among the residents aged 20 years or older in the municipality and expressed as a proportion (%). The lower the proportion, the higher the gender income inequality in the municipality (Source: Statistics Sweden).

The Statistics Sweden’s open access database was used to obtain municipal-level data on poverty in 2015, which has been calculated as the proportion (%) of persons with low economic standard in the municipality. This refers to the number of persons of all ages in the municipality who live in a household with disposable income of less than 60% of the national median disposable income.

### Statistical and geospatial analysis

For descriptive statistics, we calculated Pearson correlation (*r*) to examine whether there was a linear association between DP rate and each of the social disadvantage indicators. We examined the linearity of the associations by adding a product term (e.g. poverty*poverty) in the linear regression model including the main effect. Each association was examined separately. These analyses were performed with SAS 9.4.

Regarding geospatial analysis, we used hot spot analysis to identify spatial clusters among municipalities that had high (hot spots) or low (cold spots) prevalence of DP and social disadvantage. In this analysis, the prevalence in each municipality was analysed in the context of those of neighbouring municipalities using the Getis-Ord Gi statistic.^17^ The prevalence of, for example, DP in a municipality and its neighbours was compared proportionally to the national prevalence. An area with a high DP rate surrounded by other areas with high rates may be a statistically significant hot spot (the observed local rate of DP is higher than the expected local rate, and the difference is too large to be the result of chance alone). Similarly, an area with a low DP rate surrounded by other areas with low rates may be a cold spot.

In geospatial analysis, we first examined the location of hot and cold spots of DP among women and men and the location of hot and cold spots of each indicator of socioeconomic disadvantage. Then we examined whether a hot spot of DP was overlapping with a hot spot of any of the indicators of socioeconomic disadvantage and whether a cold spot of DP was overlapping with a cold spot of any of the indicators of socioeconomic disadvantage. ArcGIS 10.7 software was used to visualize the spatial distribution of DP prevalence, hot and cold spots of DP, socioeconomic disadvantage and overlapping hot spots and cold spots of DP and each indicator of socioeconomic disadvantage, that is, to map whether various hot and cold spots were located in the same areas in Sweden. The maps are presented with 90%, 95% and 99% confidence hot and cold spots.

## Results

The spatial distribution of age-adjusted prevalence of DP in the 290 Swedish municipalities among women and men in 2015 is shown in Supplementary figure S1. The average prevalence of DP was 6%. More women than men were receiving DP benefits at the end of 2015. Thus, we performed geospatial analysis separately for women and men and made comparisons in the relative difference in DP based on lower DP rates in the men’s hot spots than women’s.

**Figure 1:**
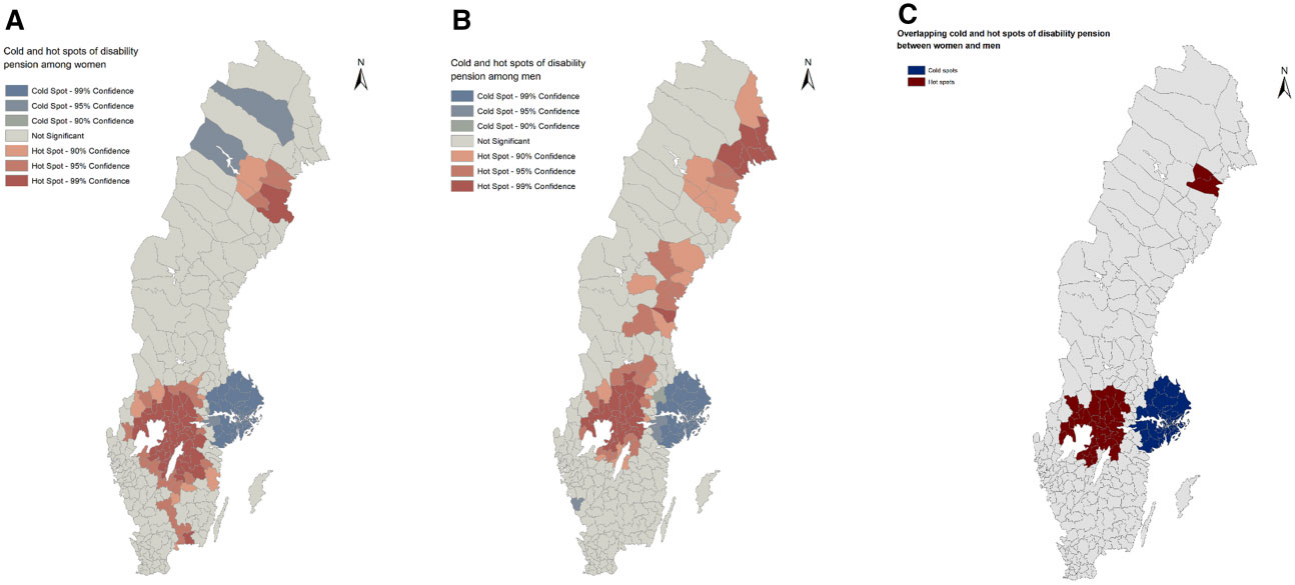
Cold and hot spots of the prevalence of disability pension among women (A) and men (B) and overlapping cold and hot spots of disability pension between women and men (C)

The results of geospatial analyses regarding clustering of DP, conducted separately among women and men, are presented in figure 1. Both municipalities of lower and those with higher than the national prevalence of DP tended to cluster. Among both women and men, there were cold spots in the metropolitan area of Stockholm, and in addition among women, there were cold spots in the northwest part of the country. Among men, there was also a small cold spot in southwest area. For both genders, hot spots were identified in the mid-south parts of Sweden, around the area of big lakes. These hot spots were larger among women, reaching toward south. Hot spots were also identified in the northeast coastal area, especially among men. Figure 1C shows whether the hot spots of DP among women and hot spots of DP among men (≥95% CI) as well as whether the cold spots of DP among women and cold spots of DP among men were overlapping, i.e. where were the rates below or above the average co-occurred. Co-occurring cold spots were observed around the area of Stockholm and co-occurring hot spots in the mid-south part of Sweden. A small overlapping hotspot was observed in northeast Sweden as well. Thus, the cold and hot spots of the prevalence of DP among women and men were in part concentrated in the same geographical areas in Sweden.

**Figure 2:**
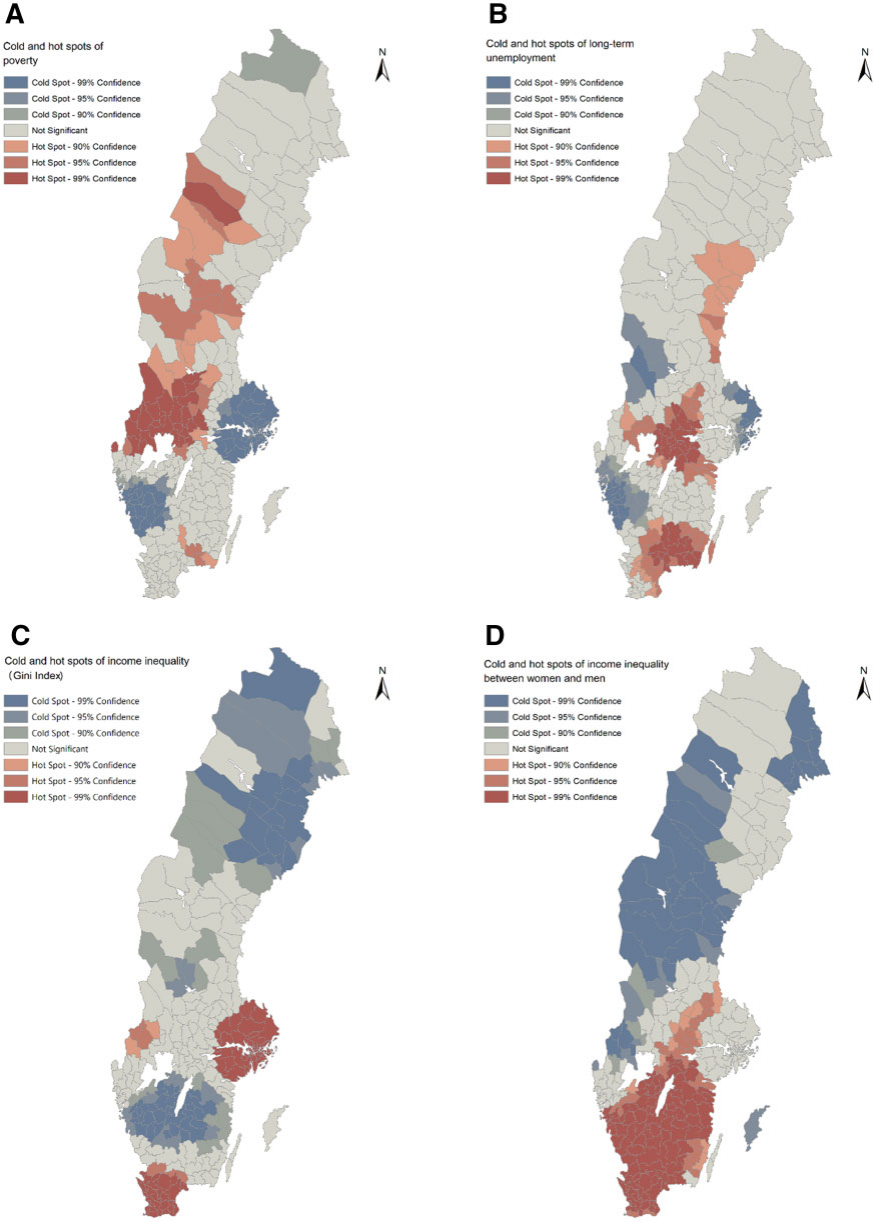
Cold and hot spots of socioeconomic disadvantage indicators across Swedish municipalities: poverty (A), long-term unemployment (B), income inequality by GINI Index (C) and gender income inequality (between women and men) (D)

We then examined whether there were geospatial cold and hot spots of each indicator of socioeconomic disadvantage in Sweden (figure 2). For poverty, large cold spots were revealed around Stockholm and Gothenburg areas, whereas hot spots with poverty higher than average were observed in a belt that covered the mid-south and mid-to-northwest parts of the country. Rather similar patterns were observed for unemployment although there were more hot spots in the south. Income inequality (GINI Index) hot spots (with the highest inequality) were observed in the wealthiest Stockholm area and in the southern Sweden. However, the indicator of gender income inequality was differentially distributed; a very large hot spot (with greater inequality) covered the whole south of Sweden, while a cold spot (with greater equality) was identified in the north.

Supplementary figure S2 shows Pearson correlations conducted separately for each indicator of socioeconomic disadvantage and the rate (%) of DP at the municipal level among women and men. All indicators were significantly associated with DP rate, except income inequality (GINI Index), which was not associated with women’s DP rate. Furthermore, there was an inverse association between GINI Index and DP rate among men. When we examined whether these associations were nonlinear, the associations of poverty, unemployment and income inequality with DP rate were significantly nonlinear; at a certain point, increasing poverty rate and unemployment rate were no longer related to increasing DP rate. However, with the GINI Index, the association was curvilinearly reverse; the higher the income inequality, the lower the DP rate and the association seemed to accelerate. For gender income equality, there was a linear negative association among women (the greater the equality, the lower the DP rate) and a weak positive association among men. Thus, the greater the share of women’s income was of men’s income in the municipality, the lower was the DP rate among women. However, there were outlier observations that could have affected the non-linear associations.

Supplementary figure S3 (women) and Supplementary figure S4 (men) show the overlapping of ≥95% confidence cold spots of DP with cold spots of each indicator of socioeconomic disadvantage and similarly, hot spots of DP with hot spots of each indicator of socioeconomic disadvantage. There were several common clusters of poverty, unemployment and gender income inequality, apart from income inequality (GINI Index) which was not overlapping with DP (figure not shown). For both genders, the high DP, unemployment and poverty rates seemed to concentrate in the mid-southern Sweden around the big lakes, while the low rates concentrated in the metropolitan of Stockholm area. Regarding gender income inequality and DP, overlapping hot spots were only identified in the southern Sweden without a clear indication of overlapping cold spots, and the hot spots were larger among women than men.

## Discussion

In the present study of geospatial distribution of DP and socioeconomic disadvantage in Sweden, we observed that among both women and men, DP cold spots were most consistently located in the Stockholm metropolitan area. For both genders, we identified large hot spots of DP in mid-south Sweden, around the area of big lakes. Higher than average DP prevalence hot spots were also observed in the northeast coastal area, especially among men. Women’s and men’s cold and hot spots were partly but not entirely overlapping, i.e. we observed that the clusters of municipalities with over- and underrepresented prevalence of women’s and men’s DP were in part concentrated in the same areas except for high DP prevalence among men being concentrated in the northeast and east. Our findings are in line with a study showing that the incidence of DP is lower in the county of Stockholm^7^ than in the rest of Sweden. Previous studies have also reported high-incidence hot spots of cardiovascular disease in northern and central Sweden,^14^ and a lower prevalence of pre-pregnancy overweight in Stockholm area and in south and south-west Sweden than in other parts of the country.^15^

Socioeconomic disadvantage was partly clustered in the same areas with DP clusters although some differences were observed. Cold spots of poverty and long-term unemployment were observed not only in Stockholm but also in southwest area, while hot spots were identified in the southern, central and eastern Sweden. Two indicators of income inequality, the GINI Index and gendered income inequality suggested somewhat contrasting geospatial distribution; the GINI Index seemed to follow the distribution of wealth in the country; greater income inequalities, i.e. greater discrepancies in income between residents of the municipality were found in the wealthiest Stockholm area, while smaller income inequalities were found in the southern and northern Sweden. A previous study carried out within one county, Stockholm, suggested a weak association between income inequality and self-rated health at the municipal level, and the association was partly explained by reduced spending on social goods in municipalities with high-income inequality.^18^ Because of these contradictory findings, more research is needed to increase understanding of geospatial distribution of income inequality and its associations with health.

The indicator of gender income inequality, in turn, suggested that the Stockholm area was neither a hot spot nor a cold spot. Instead, cold spots with higher equality were observed in large areas of north and northwest Sweden, while the whole southern Sweden was covered by hot spots with higher gender income inequality. The reasons behind these regional differences are not known but may be related to the types of economic activity in southern Sweden, such as agriculture and forest industry. However, our results also suggest that for women, greater gender inequality is a similar indicator of socioeconomic disadvantage as poverty and long-term unemployment, by being associated with DP at the local level.

### Geospatial clustering of socioeconomic disadvantage and disability pension

We found that among both women and men, the Stockholm metropolitan area emerged as a consistent cold spot (lower than average DP and lower than average socioeconomic disadvantage) while municipalities in mid-southern Sweden were identified as hot spots with overlapping socioeconomic disadvantage and DP. Receiving DP as a sole or main source of income typically reduces an individual’s economic means which may be one of the factors in the observed clustering of DP and socioeconomic disadvantage. There may be several reasons for this association. The economic life of these areas has for a long time been characterized by heavy industry, such as forest and steel industry, but their importance has decreased during the past decades, leading to higher and long-term local unemployment. Other major economic changes include increase in the high-technology industry, service and tourism sectors, although some of the areas suffer from depopulation, i.e., the younger generations with the needed skills moving to big cities, such as Gothenburg and Stockholm. High unemployment and lack of re-employment prospects for people with a reduced work capacity due to morbidity may have contributed to clustering of disadvantage and DP in these areas. However, it would be important to examine in future studies whether manual workers are affected most by structural economic changes in those areas.

A major reason for DP in Sweden is mental disorder, especially depression. A French study suggested a specific risk of depression in semi-rural regions and mid-sized urban centres in rural territories.^19^ The authors concluded that this might have been due to the lack of psychiatric healthcare resources in small towns and rural areas, a topic that should also be examined in Sweden. In addition, as the geospatial overlapping of socioeconomic disadvantage and DP was not complete, future studies would provide important knowledge by identifying areas that have low socioeconomic disadvantage despite the high DP prevalence and vice versa.

Although the main reason for differences in DP rates are differences in morbidity and type of work in relation to which the claimant’s work capacity is assessed, regional differences may also reflect differences in the implementation of the social insurance system. Major reforms have been undertaken to increase the uniformity of the social insurance system in Sweden. In 2005, the 21 local social insurance offices were centralized into one national social insurance agency in order to decrease regional differences in the assessments for fulfilling the requirements for sickness absence and DP benefits.^20^ In 2008, the eligibility requirements for DP were further tightened, requiring a permanent impairment of work capacity for those aged 30 or more. In 2015, there was a reorganization in the social insurance office resulting an even more centralized assessment system.^21^ Various sources of differences in the uniformity of social insurance have been identified.^21^ These include non-systematic differences due to, e.g. the number of handled cases as well as systematic differences arising from variations in organizational or individual decision-making. There may also be differences in how decisions are made regarding different groups of applicants, such as women and men. Furthermore, instability in the rule application over time, based for example, on the government’s initiatives, may contribute to differences in the uniformity of social insurance. Our estimate (prevalence) comprised all individual who have been granted a DP up to 2015, thus also including those who were granted DP years ago. Therefore, the regional differences observed in the prevalence of DP in our study might in part reflect the past, local praxis of DP assessment before the major reforms were applied. For example, the DP rate among men in the northeast Sweden (Norrbotten) may not reflect the current development within the area where sickness absence rates have drastically decreased during the past years.^22^ Thus, further studies are needed to investigate the effects of social insurance reforms on regional variation in DP. In addition, as the criteria for being granted DP has been tightened, more people are on long-term sickness absence, sometimes for many years. It is possible that some of those very long sickness absence periods could have been defined as DP cases.^2^

There are several limitations in this study. First, this was a cross-sectional ecological study with an inherent risk to ecological fallacy, i.e. an inference made at individual level based on aggregate data for a group. For example, the negative association observed between income inequality and prevalence of DP does not prove that higher income inequality in the municipality is causally associated with a lower probability of the residents to end up into DP. Instead, there might be a group of very wealthy residents who have an exceptionally low DP prevalence, which reduces the average DP of the municipality, and vice versa. Future studies should therefore concentrate on, e.g. Stockholm area where income inequalities are high, to get more detailed information on DP levels at neighbourhood level.^18^ Furthermore, although the DP prevalence in municipalities was age-adjusted, no other individual level factors, such as lifestyle and individual’s socioeconomic position were considered although low socioeconomic status has been associated with DP in previous studies.^7–9^ The municipal level correlation between DP and socioeconomic disadvantage may reflect individual-level correlations and our analysis of linearity may have been affected by outliers. However, the focus of our study was descriptive. We aimed to explore geospatial clustering of DP and socioeconomic disadvantage as well as consistencies and inconsistencies between them. Future studies are therefore needed to conduct a more in-depth analysis of geographical variation in DP and socioeconomic disadvantage in Sweden, such as geographically weighted regression to examine the spatial non-stationarity of the associations. Future studies might also include information about to what extent the people on DP were that for full-time or part-time. The specific strengths of our study include data that were based on reliable nationwide administrative registers, and a large number of municipalities in the analyses.

In conclusion, the present study suggests that the spatial prevalence of DP and socioeconomic disadvantage is not randomly distributed in Sweden. Our study gives additional geographic information about the link between DP and socioeconomic disadvantage, and the detailed maps may provide useful information of the phenomenon of their spatial clustering. The findings of this study provide information for the authorities to identify risk and success areas and to implement measures to prevent socioeconomic disadvantage and incapacity for work.

## Supplementary data

Supplementary data are available at *EURPUB* online.

## Ethics approval

The project was approved by the Regional Ethical Review Board, Stockholm, Statistics Sweden, the National Board of Health and Welfare and the National Social Insurance Agency, according to the Public Access to Information and Secrecy Act, the Personal Data Act and the Administrative Procedure Act in Sweden. The Regional Ethical Review Board can waive the requirement to consult in these type of large register studies and for this project stated that the consent to participate was not applicable. All register data were anonymized and de-identified and linked prior to analysis by Statistics Sweden; researchers only had access to de-identified data.

## Funding

This study was supported by the Swedish Research Council for Health, Working Life and Welfare (projects 2018-00547 and 2018-00479) and the Swedish Research Council (2017-00624). M.K. was supported by NordForsk (75021) and the Academy of Finland (311492).

*Conflicts*  *of interest*: None declared.

Key points

- Getis-Ord Gi analytical approach was used to identify geospatial cold spots and hot spots of municipalities with higher disability pension (DP)/socioeconomic disadvantage in Sweden and whether they were overlapping.
- Among women and men, cold spots of DP were most consistently located in the Stockholm area. Hot spots of DP were found in the mid-south Sweden, characterized by mid-sized urban centres in rural territories. High DP rate and socioeconomic disadvantage were geographically overlapping, except for income inequality (GINI Index).
- DP and socioeconomic disadvantage are not randomly distributed in Sweden. Further research is needed to identify preventive actions to decrease regional inequalities in work capacity.

## Supplementary Material

ckac096_Supplementary_Data
