# Supplementary material for: Clustering of disability pension and socioeconomic disadvantage in Sweden: a geospatial analysis
Source: Eur J Public Health. 2022 Jul 29;32(5):703–8. doi: 10.1093/eurpub/ckac096 (PMC9527964; doi:10.1093/eurpub/ckac096)
Supplement: ckac096_Supplementary_Data [file ckac096_supplementary_data.zip › ejph-2021-03-om-0408-File005.pdf]

Supplementary Figure 2. Pearson correlation (r) between socioeconomic disadvantage and the rate (%) of disability pension at the municipal level among women and men

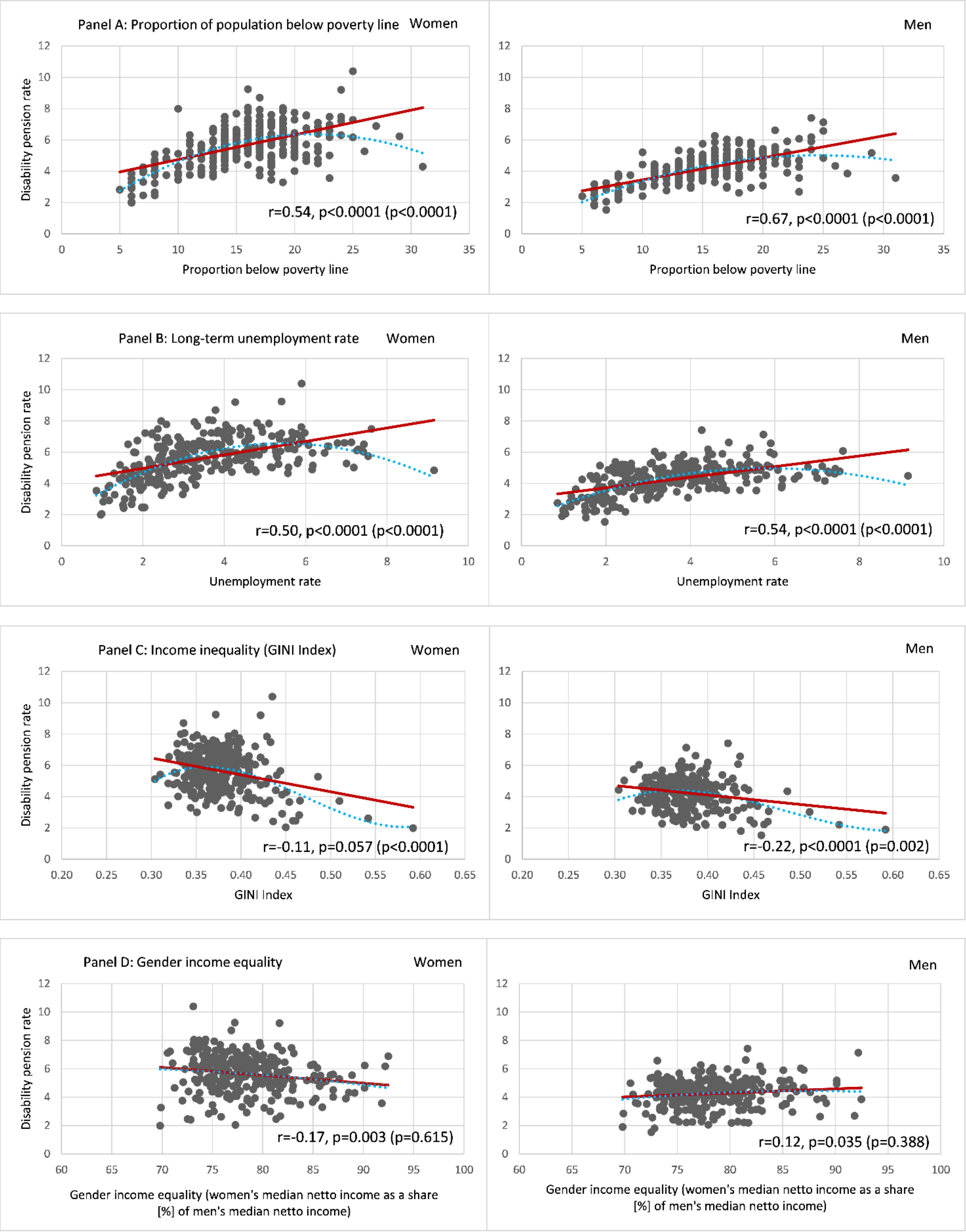

Straight line: linear association. Dotted line: nonlinear association.
P-values indicate the significance of association (P-values in parentheses indicate the significance of non-linear association).
