# Supplementary material for: Clustering of disability pension and socioeconomic disadvantage in Sweden: a geospatial analysis
Source: Eur J Public Health. 2022 Jul 29;32(5):703–8. doi: 10.1093/eurpub/ckac096 (PMC9527964; doi:10.1093/eurpub/ckac096)

Supplementary Figure 4. Overlapping cold and hot spots between socioeconomic disadvantage in the municipality and the prevalence of disability pension among men

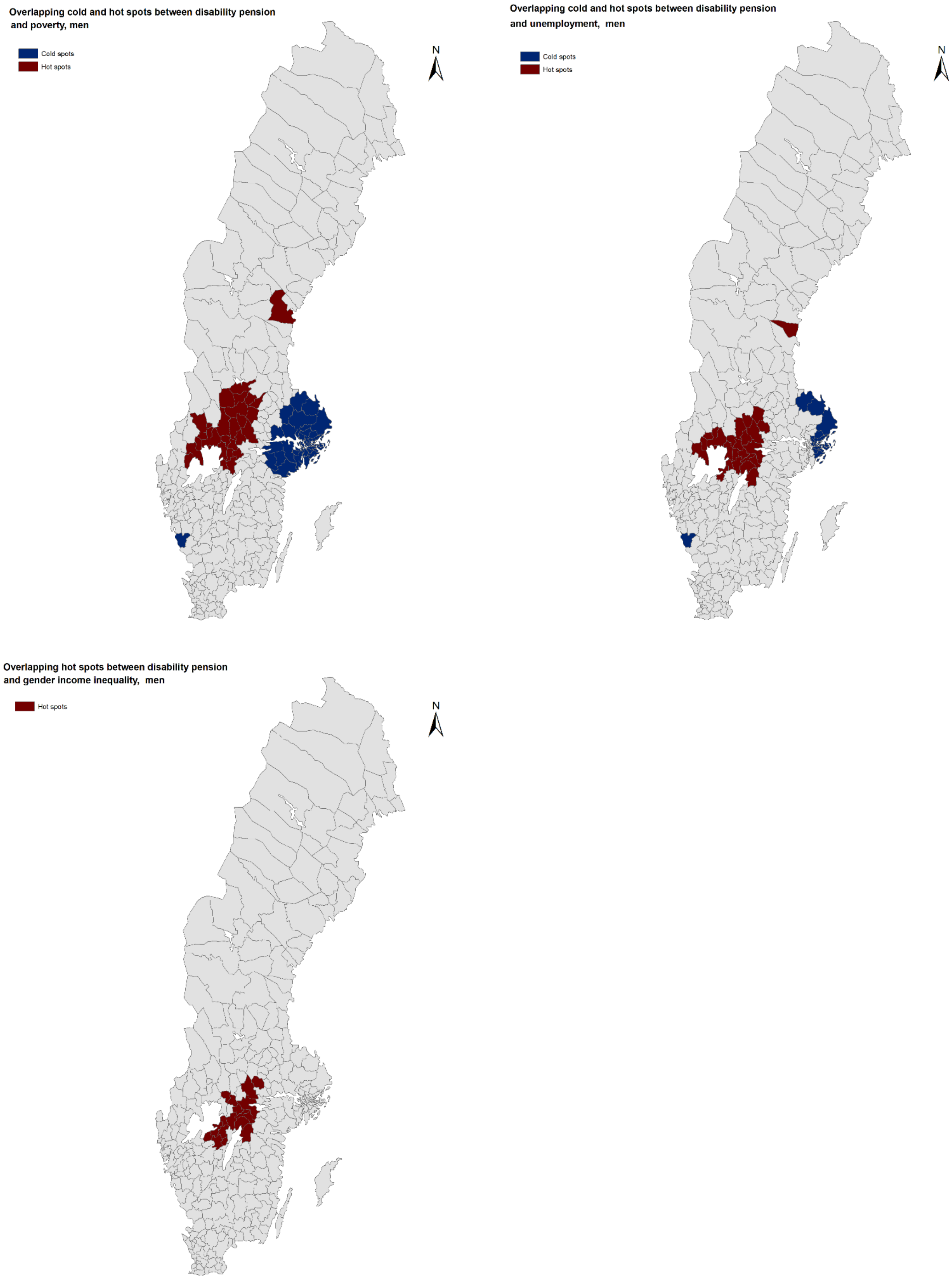

Supplement: ckac096_Supplementary_Data [file ckac096_supplementary_data.zip › ejph-2021-03-om-0408-File007.pdf]
